# Impact of A Six Week Training Program on Ventilatory Efficiency, Red Blood Cell Rheological Parameters and Red Blood Cell Nitric Oxide Signaling in Young Sickle Cell Anemia Patients: A Pilot Study

**DOI:** 10.3390/jcm8122155

**Published:** 2019-12-05

**Authors:** Marijke Grau, Elie Nader, Max Jerke, Alexander Schenk, Celine Renoux, Thomas Dietz, Bianca Collins, Daniel Alexander Bizjak, Philippe Joly, Wilhelm Bloch, Aram Prokop, Philippe Connes

**Affiliations:** 1 Department of Molecular and Cellular Sports Medicine, German Sport University Cologne, 50933 Cologne, Germany; maxjerke@googlemail.com (M.J.); a.schenk@dshs-koeln.de (A.S.); t.dietz@dshs-koeln.de (T.D.); Collins@dshs-koeln.de (B.C.); d.bizjak@dshs-koeln.de (D.A.B.); w.bloch@dshs-koeln.de (W.B.); 2 University of Lyon, University Claude Bernard Lyon 1, Interuniversity Laboratory of Human Movement Biology EA7424, “Vascular Biology and Red Blood Cell” team, 69100 Villeurbanne, France; elie.nader@free.fr (E.N.); celine.renoux@chu-lyon.fr (C.R.); philippe.joly@chu-lyon.fr (P.J.); pconnes@yahoo.fr (P.C.); 3 East Biology Centre, UF Biochemistry of Red Blood Cell Disease, Academic Hospital of Lyon, HCL, 69500 Bron, France; 4 Children’s Hospital Amsterdamer Strasse Cologne; Clinic for Children and Youth Medicine, Paediatric Oncology/Haematology, 50735 Cologne, Germany; prokopa@kliniken-koeln.de

**Keywords:** sickle cell anemia, red blood cells, red blood cell deformability, red blood cell aggregation, nitric oxide, red blood cell nitric oxide synthase, ventilatory efficiency

## Abstract

Patients with sickle cell anemia (SCA) show impaired ventilatory efficiency, altered blood rheology, high levels of oxidative/nitrosative stress and enhanced hemolysis with large amounts of circulating free hemoglobin, which reduces nitric oxide (NO) bioavailability. The aim of the study was to investigate whether physical exercise could improve these physiological and biological markers described to contribute to SCA pathophysiology. Twelve SCA patients participated in a controlled six weeks training program with moderate volume (two sessions per week with 15–30 min duration per session) and intensity (70% of the first ventilatory threshold). Parameters were compared before (T0) and after (T1) training. Daily activities were examined by a questionnaire at T0 and one year after the end of T1. Results revealed improved ventilatory efficiency, reduced nitrosative stress, reduced plasma free hemoglobin concentration, increased plasma nitrite levels and altered rheology at T1 while no effect was observed for exercise performance parameters or hematological profile. Red blood cell (RBC) NO parameters indicate increased NO bioavailability which did not affect RBC deformability. Participants increased their daily life activity level. The data from this pilot study concludes that even low intensity activities are feasible and could be beneficial for the health of SCA patients.

## 1. Introduction

Sickle cell anemia (SCA) is an inherited disease based on a modified hemoglobin gene. A single amino acid substitution—glutamic acid changed to valine—at position six of the β-chain of hemoglobin results in an abnormal hemoglobin S (HbS). SCA is evidenced as homozygous for this mutation (HbSS). HbS polymerizes under low oxygen tension [1] which affects the structure of red blood cells (RBCs). Normal shaped RBCs are biconcave but polymerization of HbS results in calcium influx which leads to dehydration and finally sickling of the RBCs [2].

Sickle RBCs show reduced capacity to deform which affects blood flow through the microcirculation and tissue perfusion. Sickle RBCs are also very fragile and RBC lifespan is highly reduced [3] by about seven times. Lyzed RBCs release cell-free hemoglobin into the blood stream which binds to nitric oxide (NO) and thus reduces the availability of this important cell messenger. The reduction of bioavailable NO but also the depletion of L-arginine, the substrate for NO production, oxidative stress and endothelial inflammation caused by free heme [4] results in vascular dysfunction. This vascular dysfunction, in combination with the alterations in RBC rheology, increases the risk for frequent painful vaso-occlusive events [5,6] In addition, enhanced hemolysis leads to the development of severe chronic organ damages in SCA [7]. Thus, according to a study by Lanzkron et al. [8], the life expectancy of SCA patients is significantly reduced compared to healthy people, and was found to be 42 years for females and 38 years for males. 

Physical activity and endurance exercise have been proven to be beneficial for mental and physical health and were reported to reduce the risk for certain diseases like diabetes, cancer, coronary heart disease or mental health problems (for a review, see [9]). Besides the positive effects on complex metabolic processes, endurance exercise was also reported to have a high potential to positively affect RBC rheology [10,11,12,13] SCA patients show a reduced exercise tolerance because of several factors including reduced oxygen carrying capacity related to low hemoglobin concentration, pulmonary vascular disease, peripheral vascular impairments and alterations in RBC rheology (for a review, see [14]). However, physicians are often reluctant to promote regular physical activity in SCA patients [14,15,16] The main reason is that the metabolic changes occurring during exercise may promote HbS polymerization, RBC sickling, oxidative stress and inflammation, and ultimately lead to vaso-occlusive crises. Nevertheless, several studies showed that a moderate exercise of 10–20 min duration is well tolerated by both adults and children with SCA and does not cause further alterations in RBC rheology, hemolysis or inflammation [17,18,19,20,21,22] In addition, recent works in transgenic sickle cell mice (SAD and Townes models) showed that regular physical activity decreased oxidative stress and inflammation, and improved blood rheology [23,24,25,26]. These data support the fact that SCA patients are able to exercise [27] and that regular moderate physical activity could be beneficial for their health. Reports on accurate training programs in SCA patients are missing to date and it is difficult to know what kind of training programs could be prescribed without any risk. Thus, the aim of the study was to investigate for the first time the effect of a six week training program on a bicycle ergometer at defined intensity and volume on ventilatory efficiency, RBC rheology, RBC NO and oxidative stress parameters in young SCA patients. Moreover, questions on daily activity level were asked before training and one year after the end of the study which aims to determine whether the training programs motivates the participants to increase daily activity levels.

## 2. Experimental Section

### 2.1. Study Protocol

The protocols used in this study were reviewed and approved by the ethics committee of the German Sport University Cologne (date of approval: 16th July, 2014). The protocols are in line with the Declaration of Helsinki and all participants or legal guardians of the participants gave written informed consent to participate in this study. A total of 12 young SCA patients (4m/8f) were included in this study (fetal hemoglobin (HbF) = 15.4 ± 9.7%, HbS = 77.9 ± 14.3%). Anthropometric data were as follows (mean/median/standard deviation): 13.4/13.0/6.4 years, 1.5/1.5/0.2 m, 38.3/38.5/14.5 kg, 17.3/17.1/2.4 kg/m^2^. SCA diagnosis was made by hemoglobin electrophoresis and confirmed by genetic studies in case of unclear electrophoresis results. Patients were at steady-state (i.e., without any acute vaso-occlusions or hospitalized complications and no blood transfusion in the last three months prior to intervention). Seven patients received hydroxycarbamide medication at the beginning of the training program (18–40 mg/kg body weight) and nine patients received hydroxycarbamide medication at the end of the training program (12–40 mg/kg body weight). Medication of the two additional patients with hydroxycarbamide during the protocol started in the first days after the beginning of training. 

### 2.2. Definition of the Training Program

SCA patients performed a progressive submaximal exercise test on a cycle ergometer (ergoselect 150, ergoline, Bitz, Germany) with breath-by-breath gas exchange analysis (Metalyzer 3B, Cortex Biophysics GmbH, Leipzig, Germany). A height adapted protocol was used (similar to [28]) to ensure that initial workload and workload increments were comparable between the subjects which were of different age and thus height varied between 1.05 m and 1.70 m. In detail, the measurement started with a 2 min breath-by-breath measurement at rest. The initial workload and workload increments were based on height of each subject: 10 watts for height <1.20 m, 15 watts for 1.20–1.50 m, and 20 watts for >1.50 m. Watt power increased every 2 minutes. A cadence of 60 revolutions per minute (rpm) was maintained. The measurement was stopped when the subject reached the first ventilatory threshold (VT 1), which defines the switch from aerobic to aerobic–anaerobic metabolism (=T0). The presence of VT 1 was then verified by published methods [29]. Recorded exercise parameters included VT 1 [ml/min/kg], time to (reach) VT 1 [s] and power at VT 1 [W]. The ratios ventilation/oxygen uptake (VE/VO_2_)—which is the ratio of the volume of gas expired per minute to the volume of oxygen consumed per minute—and ventilation/carbon dioxide production (VE/VCO_2_)—which refers to the liters of ventilation per liter of CO_2_ output—were calculated at rest and at every step of the exercise test [30]. These ratios give information on the ventilatory efficiency of the subjects with normal values being around 30 for normal and reaching higher values in patients with chronic disease such as sarcoidosis (values around 60), chronic obstructive pulmonary disease (values around 50–60) or dilated cardiomyopathy (values around 40) [31]. The participants started the training program one week after this exercise test. The training sessions were conducted on a bicycle twice per week with training intensity at 70% VT1. The patients were asked to keep up a cadence of 60 rpm at all time. Training volume increased from 15 min during weeks 1 and 2 to 20 min during weeks 3 and 4, and 30 min during weeks 5 and 6, respectively. Training was scheduled on Mondays and Thursdays. SCA patients performed a second exercise test, which was comparable to the one performed at T0, one week after the end of the training program (T1). Additionally, participants were asked to fill out a questionnaire at T0 which was adapted from the Motoric-module-questionnaire (MoMo-AFB) [32] to determine the daily activity state of the participants. The questionnaire was repeated one year after the end of the study to examine whether the training intervention encouraged the participants to further increase daily activities.

### 2.3. Blood Sampling

Venous blood was sampled from the elbow vein at rest before the exercise test at T0 and T1, respectively, and anticoagulated using sodium heparin (BD; Heidelberg, Germany) or EDTA (BD; Heidelberg, Germany). EDTA blood was used to measure basal RBC parameters. The remaining sample was directly stored at −80 °C for measurement of the different hemoglobin fractions using capillary electrophoresis (Capillarys 2 Flex Piercing Sebia, Evry, France). Sodium heparin anticoagulated whole blood was used to measure RBC deformability and RBC aggregation (described below). The remaining sample was separated by centrifugation at 3600 g for 2 min and 4 °C. The plasma supernatant was used for the measurement of plasma free hemoglobin and plasma nitrite levels and were immediately stored at −20 °C and −80 °C, respectively, until measurement. An RBC pellet was processed as described for the different parameters (see below), snap frozen and stored at appropriate temperature until measurement.

### 2.4. Basal RBC Parameters

Basal RBC parameters including hemoglobin concentration (Hb), mean corpuscular volume (MCV), mean corpuscular hemoglobin (MCH) and mean corpuscular hemoglobin concentration (MCHC) were directly determined in whole blood using the hematology analyzer Sysmex Digitana KX-21N (Sysmex, Switzerland).

### 2.5. Plasma Free Hemoglobin Concentration

Plasma samples were thawed on ice and the Human Hemoglobin ELISA kit (Abcam, Cambridge UK) was applied to measure the free human hemoglobin concentration according to the instructions supplied by kit booklet.

### 2.6. RBC Deformability

Whole blood was mixed with a polyvinylpyrrolidone solution in a 1:250 ratio (PVP; 28 cP at 37 °C, RR Mechatronics, Hoorn, The Netherlands) and RBC deformability was measured by ektacytometry (laser assisted optical rotational cell analyzer; LORCA; RR Mechatronics, Hoorn, The Netherlands) [33]. The samples were sheared in a Couette system at nine consecutive shear stresses ranging from 0.3 and 50 Pa. A laser beam was directed through the samples. Deformation of RBCs upon shear stress affected the diffraction pattern of the laser beam which was measured by the LORCA software and used to calculate an elongation index which is presented herein. Thus, a higher elongation index represents greater RBC deformability.

### 2.7. RBC Aggregation

RBC aggregation was measured at 37 °C by syllectometry using the LORCA system. As recommended [34], prior to RBC aggregation measurement, all samples were fully oxygenated for 15 min with the use of a Roller Mixer (Karl Hecht KG, Sondheim vor der Rhön, Germany). Oxygenated samples were transferred to the Couette system and changes of backscattered light were recorded over 120 sec using two photodiodes and presented as a graph (syllectogram) to calculate an aggregation index (AI%). To demonstrate the threshold shear rate balancing RBC aggregation and disaggregation, an iteration procedure was then performed to primarily calculate dIsc min. This parameter defines the minimum change in backscatter intensity during the iteration procedure, representing the minimum shear rate where RBC aggregates start to disaggregate (y at dIsc min (s^−1^)).

### 2.8. RBC L-Arginine 

L-arginine concentration of RBCs was measured using the L-arginine ELISA Kit (Immun Diagnostik AG, Bensheim, Germany) according to the manufacturers´ instructions. Frozen RBCs were lysed for 20 min in an ultrasound bath. Samples were centrifuged at 21,000 g and 4 °C for 10 min and the supernatant was used for the analysis [35]. The results were evaluated using a 4-parameter-algorithm with L-arginine concentration being inverse proportional to color development.

### 2.9. Total RBC- NO synthase (RBC_NOS) and Akt kinase, Activation State of RBC-NOS and Akt Kinase and Nitrotyrosine Staining

For immunohistochemical staining of RBC proteins and protein activation state, RBC pellet was fixed in 4% formaldehyde [36]. Blood smears were prepared and heat fixed. A test and a control area were marked on each slide using a grease pen for inner-slide-control. Both areas were washed with tris-buffered saline (0.1 mol tris-buffered-saline (TBS), pH 7.6) and incubated with 0.1% trypsin solution. RBCs were then treated with a solution containing 2% hydrogen peroxide, 80% methanol, rest aqua dest and non-specific antibody binding was minimized by following incubation of both areas with 3% skim milk. The test area of each slide was incubated with the respective primary antibody in a 0.3% skim milk solution: Anti-eNOS/NOS Type III (dilution: 1:700, BD, Heidelberg, Germany), Anti-phospho eNOS (Ser1177) (dilution: 1:150, Millipore, Burlington, USA), Phospho- Akt (Ser473) (dilution 1:500, Cell Signaling, Danvers, USA), Anti-Akt1/PKBα (dilution 1:500, Merck-Millipore, Burlington, USA) and Anti-Nitrotyrosine (dilution 1:500, Upstate/Millipore, Burlington, USA). Slides were washed with TBS, treated with 3% normal goat serum (Dako, Glostrup, Denmark) and incubated with a secondary goat anti-rabbit antibody (dilution: 1:400, Dako, Glostrup, Denmark). 3,3-diaminobenzidine-tetrahydrochloride (DAB) solution (Sigma-Aldrich, St. Louis, USA) in TBS was used to develop staining. Slides were then dehydrated by exposure to alcohol solutions of increasing concentration and sealed using Entellan® (Merck, Darmstadt, Germany). Images were taken from the stained slides using a Zeiss microscope coupled to a CCD-camera (DXC-1850P, Sony, Berlin, Germany). The semi-quantitative analysis of the grey values was conducted using the ‘Image J’ software (National Institutes of Health, Bethesda, USA). Grey values of a total of 50 RBCs from at least four images were determined in the test area and subtracted from the background value, which was measured in a cell-free area of the slide in order to obtain staining intensity caused by the binding of the antibodies. Then, the grey values of a total of 10 RBCs from at least two images were determined in the control area and also subtracted from the background value to obtain the baseline grey values of unstained RBCs. Finally, grey values of RBCs from test and control areas were subtracted to obtain net staining intensities.

### 2.10. Plasma Nitrite and RBC Nitrite/RSNO/Fe-NO 

Nitrite is produced by the oxidation of NO and 90% of the plasma nitrite concentration is derived from the L-arginine/NO pathway [37]. Thus, measurement of plasma nitrite represents a reliable marker for endothelial NO synthase activity and NO concentration [38]. Plasma samples were thawed on ice and immediately measured. After separation of whole blood, RBC pellet was mixed with a nitrite preservation solution, mixed, snap frozen and stored at −80 °C until measurement [39,40]. Plasma nitrite and RBC Nitrite/RSNO/Fe-NO concentrations were measured as described earlier [37,38]. Thawed RBC samples were mixed with ice-cold methanol (VWR International, Darmstadt, Germany), centrifuged and the Nitrite/RSNO/Fe-NO levels of the supernatant were measured. The sample was injected into an acidified tri-iodide (potassium iodide and iodine in acetic acid) solution and this NO was analyzed using a chemiluminescence NO detector (CLD 88e, EcoPhysics, Munich, Germany). All samples were measured in triplicate. The Chart FIA software (EcoPhysics, Munich, Germany) was used to integrate the area under the curve and the sample Nitrite/RSNO/Fe-NO concentration was calculated by the use of standard solutions. RBC Nitrite/RSNO/Fe-NO concentration of the samples was corrected for Nitrite/RSNO/Fe-NO concentrations of methanol and preservation solution, respectively.

### 2.11. RBC Lipid Peroxidation

An RBC pellet was diluted with 0.1 mol PBS (pH 7.4) to obtain 5 × 10^7^ RBCs/ml, snap frozen and stored at −80 °C. Measurement of lipid peroxidation was performed by using the TBARS Assay Kit (Cayman Chemical, Ann Arbor, USA) according to the protocol supplied by the kit booklet. 

### 2.12. RBC Total Antioxidant Capacity

RBC pellet was diluted with 0.1 mol PBS (pH 7.4) to obtain 1 × 10^7^ RBCs/ml, snap frozen and stored at −80 °C until measurement. Samples were thawed on ice and the total antioxidant capacity assay kit (Abcam, ’Cambridge, UK) was applied to measure RBC total antioxidant capacity according to the manufacturers´ instructions. 

### 2.13. Statistics

Statistical analyses and presentation of data were conducted using commercial software (Prism, GraphPad Software Inc., San Diego, USA). All data are presented as mean ± standard deviation (SD). Normal distribution of the data was tested using D´Agostino and Pearson omnibus normality test. Comparison of data was done using paired t-test or a two-way repeated measures ANOVA when appropriate, followed by a Bonferroni post hoc test. 

## 3. Results

### 3.1. Performance Parameters

**Figure 1:**
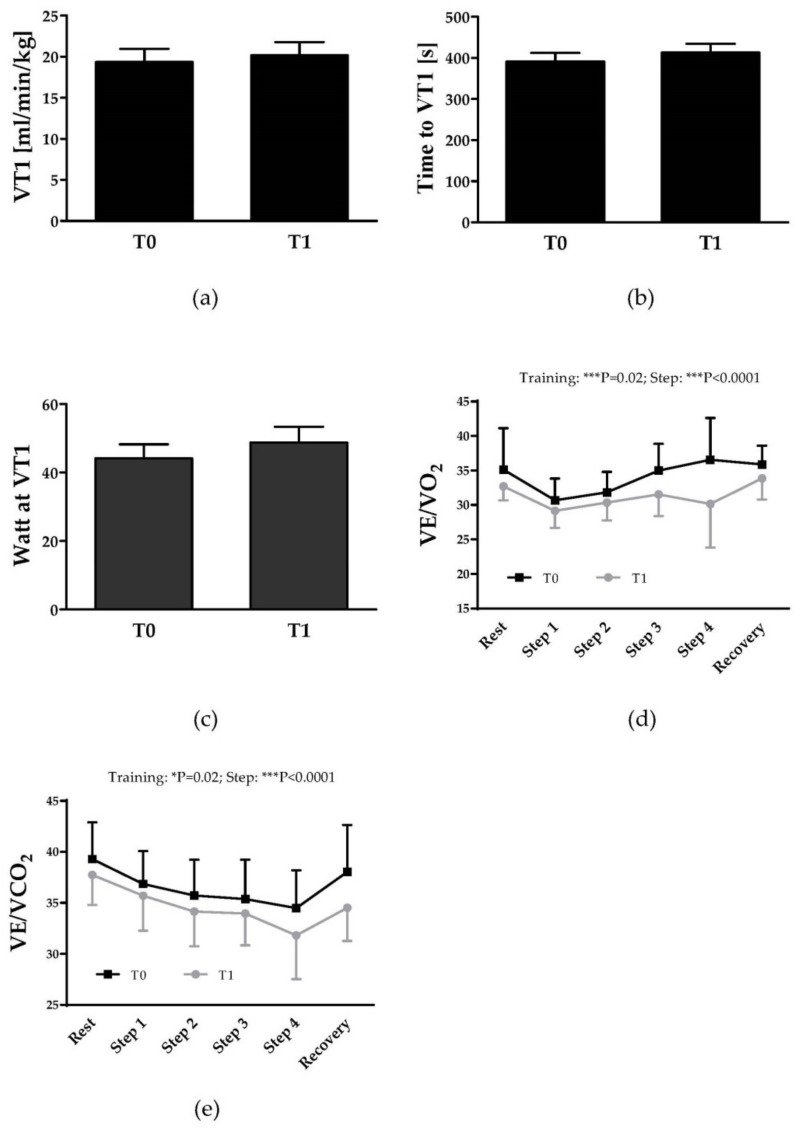
Performance parameters and ventilatory efficiency of young SCA patients during the exercise test at T0 and T1. (**a**) First ventilatory threshold (VT1), (**b**) time to VT1 and (**c**) watt power at VT1 showed no statistical difference between T0 and T1, respectively. For both (**d**) VE/VO_2_ and (**e**) VE/VCO_2_, we observed a training (*p* = 0.02) and a step effect (*p* < 0.0001). SCA, sickle cell anemia.

Tested performance parametes including VT1, watt power at VT1 and time to VT1 showed no significant difference between T0 and T1, respectively (Figure 1a–c). 

### 3.2. Ventilatory Efficiency

VE/VO_2_ and VE/VCO_2_ represent the ventilatory equivalent for O_2_ and CO_2_, respectively, and describe the relation between minute ventilation and oxygen consumption/carbon dioxide production. Ventilatory equivalents significantly decreased from T0 and T1. Two-way repeated measure ANOVA revealed a significant training (p = 0.02/p = 0.02) and step (p < 0.0001/p < 0.001) effect for both VE/VO_2_ and VE/VCO_2,_, respectively (Figure 1d,e). 

### 3.3. Blood Parameters and Free Hemoglobin Concentration

Blood parameters showed no significant difference between T0 and T1. Free hemoglobin concentration significantly decreased (*p* < 0.01) at T1 (Table 1). 

### 3.4. Red Blood Cell Rheology

**Figure 2:**
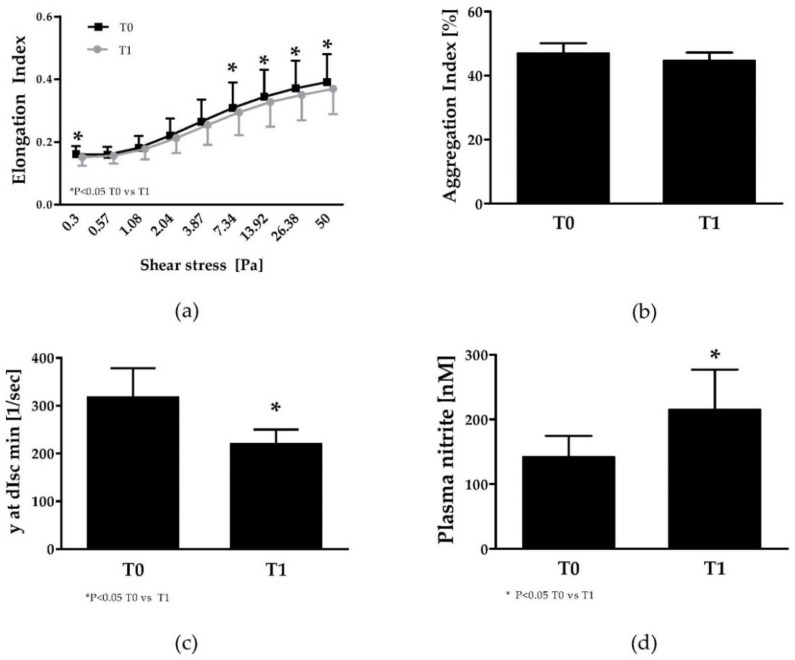
Red blood cell (RBC) rheological and plasma nitrite parameters of SCA patients at T0 and T1: (**a**) RBC deformability. **(b)** Aggregation Index, (**c**) RBC disaggregation threshold (i.e., the strength of RBC aggregates) and (**d**) plasma nitrite. Significant difference between T0 and T1: * *p* < 0.05.

RBC deformability values are presented for the nine measured shear stresses in Figure 2a. RBC deformability decreased at T1 with significantly decreased values measured for 0.3 Pa (*p* = 0.0202), 7.34 Pa (*p* = 0.0395), 13.92 Pa (*p* = 0.0418), 26.38 Pa (*p* = 0.0285) and 50 Pa (*p* = 0.0285), respectively. Percent change of RBC deformability from T0 to T1 was very slight: −5.95% at 0.3 Pa, −2% at 0.57 Pa, −1.84% at 1.08 Pa, −3.67% at 2.04 Pa, −4.22% at 3.87 Pa, −4.51% at 7.34 Pa, −4.84% at 13.92 Pa, −5.58% at 26.38 Pa and −5.39% at 50%. These changes are too low to have any physiological impact [41]. Aggregation Index (AI) slightly decreased from T0 to T1 (*p* = 0.2). Minimal shear rate needed to dissociate RBC aggregates (y at dIsc min) significantly decreased (*p* = 0.014) from T0 to T1 (Figure 2b,c). 

### 3.5. Plasma Nitrite Concentrations

Plasma nitrite concentration significantly increased from T0 to T1 suggesting that training increased either endothelial NO production or NO bioavailability (Figure 2d).

### 3.6. Red Blood Cell Nitric Oxide Signalling

**Figure 3:**
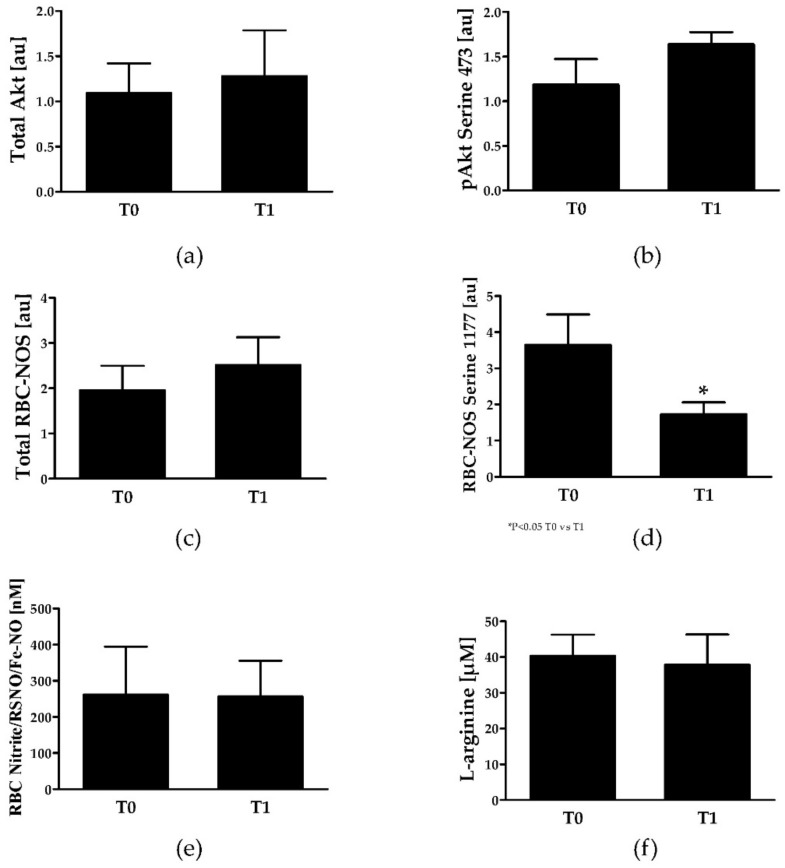
RBC NO signaling parameters in SCA patients at T0 and T1. (**a**) RBC total Akt kinase staining, (**b**) activated Akt kinase, reflected by phosphorylation of serine 473 residue. (**c**) Total RBC-NOS staining, (**d**) Activation of RBC-NOS, reflected by phosphorylation of serine 1177 residue, (**e**) RBC nitrite/RSNO/Fe-NO concentration and (**f**) RBC L-arginine concentration remained unaltered during intervention. Significant difference between T0 and T1: * *p* < 0.05.

Total Akt kinase signal but also phosphorylation of Akt kinase did not change during intervention (Figure 3a,b). The total RBC-NOS signal remained unaffected by intervention but activation of RBC-NOS, reflected by phosphorylation of RBC-NOS serine 1177 residue, decreased from T0 to T1 (*p* = 0.03) (Figure 3c,d). RBC nitrite/RSNO/Fe-NO concentration was not affected by intervention and L-arginine concentration within RBCs also remained unaltered (Figure 3e,f).

### 3.7. Oxidative Stress Parameters

**Figure 4:**
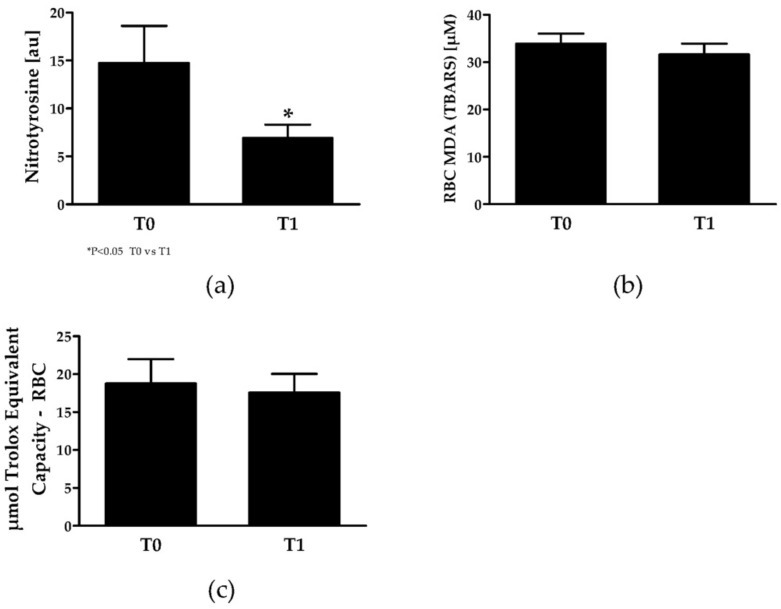
RBC oxidative stress marker in SCA patients at T0 and T1. (**a**) RBC nitrotyrosine signal, (**b**) RBC MDA and **(c)** total antioxidant capacity. Significant difference between T0 and T1: * *p* < 0.05. MDA, malondialdehyde.

Nitrotyrosine is a product of tyrosine nitration and marker for cell damage. NO and superoxide anions react to form peroxynitrite which is capable of nitration of tyrosine residues. Nitrotyrosine staining significantly decreased from T0 to T1 (*p* = 0.04) (Figure 4a). RBC malondialdehyde (MDA) levels (Figure 4b), a marker for lipid peroxidation, and total RBC antioxidant capacity (Figure 4c) were not affected by training (Figure 4b). 

### 3.8. Considerations About HU Treatment

Although the size of the subgroups (i.e., patients without HU (*n* = 3), patients with HU at both T0 and T1 (*n* = 7) and patients starting HU 1–2 weeks after T0 (*n* = 2)) was too small to perform statistical analyses, we looked at the possibility of different behavior regarding the different parameters analyzed. As expected, MCV and Hb were higher in the patients who were under HU therapy before the training period (100.4 ± 9.1 fl and 9.9 ± 2.7 g/dL, respectively) compared to patients not receiving HU at T0 (80.5 ± 10.0 fl and 8.3 ± 0.9 g/dL, respectively). However, the lack of training effect on these parameters for the whole group was also observed for each of the subgroups. The effect of training on free hemoglobin level was observed for the three groups but seems to be more pronounced in the HU treated groups. Because HU improves RBC deformability in SCA [42], it was not surprising to find higher EI values in the HU treated patients compared to those not receiving HU. However, the slight decrease occurring with training was of the same magnitude in the three groups. In addition, NO levels were higher in the HU treated patients; probably because HU is a NO donor [43], but training effect was similar in the three subgroups. Thus, the increase in plasma nitrite after exercise is comparable between the whole study population and the respective subgroups and not caused by varying HU therapy. Finally, VT1 remained unchanged in the whole group after training but we noted slight increment in the non-HU patients between T0 and T1. This is in agreement with the fact that the effects of training on VE/VO_2_ and VE/VCO_2_ was a little bit more pronounced in the non-HU group compared to the two other groups. The other parameters did not really differ between the three subgroups and the training effect seemed to be similar.

### 3.9. Questionnaire

The WHO documents on daily physical activity for age group 5–17 years recommend daily moderate- to vigorous-intensity physical activities for at least 60 minutes and muscle strengthening activities 2–3 times per week. Physical activites not only include sports but also play, transportation (to school, for example), planned exercise, school activities and recreation [44]. Evaluation of the questionnaire on daily activities revealed that, prior to the start of the training intervention described herein, activity level of SCA patients is low compared to the WHO suggestions. SCA patients were active on 1.4 ± 0.9 days per week. In total, 60% of SCA patients participated in school sport activities and described moderate to heavy sweating and shortness of breath during sport. Interest in sport was moderate. In total, 20% of the SCA patients reported dizziness, pain or tiredness after recreational exercise. In total, 40% reported that they were hospitalized after recreational exercise. In total, 100% of the SCA patients go to school by bus or train. The same questions were asked one year after the end of the training program and 80% report that they increased their daily activities after the training program. The results reveal a trend through an increase in active days per week to 3.0 ± 2.5 but statistical significance was not reached (*p* > 0.05) and children described only moderate sweating and sometimes shortness of breath during sport. In total, 40% of the SCA patients reported dizziness or tiredness after exercise. None of them were hospitalized after recreational exercise. In total, 80% of the SCA patients went to school by bus or train and 20% walk. None of the patients included in this study were hospitalized during the training phase or in the short term after each physical session.

## 4. Discussion

To the best of our knowledge, this is the first study describing the effects of a six week training program on ventilatory efficiency, RBC rheology, RBC NO and oxidative stress parameters in young SCA patients. Key findings of this investigation suggest an improvement in ventilatory efficiency because of lower VE/VO_2_ and VE/VCO_2_ values after the training, a reduction in free hemoglobin concentration at rest, a reduction in nitrosative stress marker and an overall increased daily life activity level determined by questionnaires. The training program had no deleterious effects on RBC rheology and RBC NO signaling.

Analysis of the questionnaire on daily activities revealed that SCA patients are interested in participating in sports activities but feel very insecure on doing so. They avoid physical activities during their daily routine. For example, they use a bus or train to get to school although alternatives (walking, taking the bicycle) would be possible. A follow up questionnaire one year after the end of the training program revealed a trend through an increase in daily activities and hospitalization rate after recreational physical activities decreased, though, more patients were under hydroxyurea therapy. In general, daily activities could be further increased but need additional guidance. Liem et al. [45] showed that 77% of 13 children with SCA completed 89% of prescribed sessions at home without any adverse events. However, the authors noted that adherence to prescribed training sessions decreased in the second half of their program, suggesting that higher efforts need to be made to motivate SCA to practice physical activities on a regular basis. The increase in activity levels might be beneficial for the health of SCA patients [21,46] but a higher number of patients involved in such a training program is needed to better answer this question.

Very few studies, and most of them time case studies with poorly controlled exercise intensities and volumes, investigated the effects of regular physical activity in SCA [46,47]. Tinti et al. [46] previously reported that two sessions of 45 min per week of aquatic rehabilitation decreased chronic pain, improved respiratory muscle strength and quality of life in a SCA patient. Alcorn et al. [47] demonstrated that exercise therapy mixing moderate strength and endurance exercise, recreational gymnastics, stationary bicycle riding and games for 10–30 min duration decreased the length of hospitalization with vaso-occlusive crisis in SCA children. More recently, a well-controlled study showed that individualized program with three calibrated (i.e., intensity corresponded to a blood lactate concentration of approximatively 2.5 mM) 45 min exercise sessions per week, for 8 weeks, was safe for adults with SCA and was able to increase functional capacity (i.e., an increase of power output measured at 4 mM of blood lactate concentration) [21]. However, none of these studies investigated the changes in the different biological markers and modulators of the clinical severity in SCA.

As previously described, specific performance parameters including watt power at VT 1, time to VT 1 and VO_2_ at VT 1 were lower in SCA patients compared to healthy controls [18,19]. The ventilatory threshold is considered a reliable marker to assess cardiorespiratory fitness and is defined as the point during exercise where pulmonary ventilation begins to disproportionately increase with regard to the increase in oxygen uptake. Thus, the VT 1 defines the onset of anaerobic metabolism contributing to ATP production [48]. Endurance training is supposed to delay VT 1 [48,49] but the data presented herein remained unaltered during training, suggesting that the chosen intensity, volume or both were too low to have an effect on the performance parameters. In contrast, the parameters of ventilatory efficiency (VE/VO_2_ and VE/VCO_2_) were significantly lower after training both at rest and during exercise compared to before. The VE/VCO_2_ index defines ventilatory efficiency, for it reflects the interaction between pulmonary ventilation, pulmonary perfusion, and cardiac output, contributing to the prognosis of the patient. In patients with cardiac diseases, elevated VE/VCO_2_ index indicates inefficient ventilation [50]. VE/VCO_2_ moreover provides information about the ventilatory demands to remove produced CO_2_. Normal values are in the range of 25–30 [51] and higher levels as presented for SCA herein and elsewhere [19], are a marker of inefficient ventilation and poor gas exchange which can be due to hyperventilation or increased dead space. Although this study design should be characterized as pilot study and although study populations seem to be somewhat heterogeneous because some were under hydroxyurea therapy the whole time, and some received hydroxyurea during the intervention and others did not receive hydroxyurea treatment, the overall reduction of the values during training suggests that although the training program might be too low to affect aerobic performance capacity, ventilatory efficiency was improved which means that less liters of ventilation were needed to eliminate metabolically produced CO_2_ and less liters of ventilation were needed per liter of oxygen consumed [52]. These adaptations could considerably improve the ability of SCA children to perform regular exercise and limit their exercise intolerance. Future studies should differentiate between hydroxyurea treated and non-treated patients because it seems that the effect of training might be higher in non-treated patients. Furthermore, future studies should extend the study period and maybe add more training sessions per week for a higher outcome.

Repeated cycles of RBC sickling and unsickling damage the cell membrane and increase cell fragility which finally undergo hemolysis. SCA patients show levels of plasma free hemoglobin concentration of about 6 mg/dl at normal phases which is up to 20 times higher compared to healthy people (0.3 mg/dL) [53] and approximately 4 µM heme compared to 0.2 µM heme in healthy controls [54]. Any events that would increase the rate of RBC sickling-unsickling cycles, such as those which occurs during exercise (metabolic acidosis and increased free radical production for instance), could increase the risk for cell lysis. Although the plasma free hemoglobin levels reported herein were slightly lower compared to the cited studies, our results demonstrated a decrease of plasma free hemoglobin after training. Decreased levels of plasma free hemoglobin would increase NO bioavailability and indeed, plasma nitrite levels were significantly higher at T1 compared to T0. It remains speculative whether this might also be related to increased eNOS activity because this was not measured. Comparing plasma and RBC NO parameters indicates that the training intervention did not affect total RBC Akt kinase levels or phosphorylation, thus activation, of Akt kinase at serine 473. Furthermore, total RBC-NOS content remained unaffected but phosphorylation of RBC-NOS serine 1177 and thus basal activation of RBC-NOS was lower at T1 compared to T0. Functional activity of RBC-NOS has been described by several authors [55,56,57,58] and RBC-NOS produced NO was described to represent an important signaling molecule positively affecting RBC deformability [57,59]. Although RBC-NOS activation decreased during training, the substrate for NO production—L-arginine—but also the levels of the resulting product—RBC nitrite/RSNO/Fe-NO—remained unaffected by the training program. RBC-NOS activation was shown to be higher in SCA patients compared to healthy controls [60] and a recent study on acute exercise effects on RBC-NOS activation in young patients with SCA also indicates a reduction in RBC-NOS activation by exercise [18]. Given the fact that RBC NO levels were comparable between T0 and T1, although RBC-NOS activation was reduced, suggests that NO bioavailability increased by other factors and that reduction in RBC-NOS activation represents an adjustment mechanism because of changed NO availability.

Within the cell, NO reaction routes are diverse. NO can either be oxidized to nitrite and nitrate or reacts with hemoglobin or superoxide to form peroxynitrite, which would limit NO availability (see [61]). SCA patients show higher levels of reactive oxygen and nitrogen species compared to healthy controls [62]. Data of the present study showed reduced nitrotyrosine levels within the RBCs after training suggesting an increase in NO availability because of a reduction of nitrosative stress parameters. Training intervention did not affect lipid peroxidation or total antioxidant capacity which, like for other parameters, might be explained by a too low training intensity and volume. 

Surprisingly, we observed a significant decrease of RBC deformability at some shear stresses after training. RBC deformability depends on the surface area to volume ratio, structural properties of the cytoskeleton, the interaction between the cytoskeleton with the membrane, intracellular viscosity and NO availability (for a review, see [63]). Although not significant, MCHC slightly increased after training, which could partly explain the decrease in RBC deformability. However, the magnitudes of changes in RBC deformability were small (less than 6%) and are unlikely to have an effect in vivo [41]. Differences measured ex vivo > 10% are suggested to have a physiological effect. Changes in MCHC might also be related to the timing of training intervention and it seemed that training rehabilitation occurred during summer and that patients could have been more or less hydrated from one visit to another. Thus, for future studies is might be important to thoroughly control hydration status of the participants. Moreover, the decrease in plasma free hemoglobin concentration after training does not support that RBCs were more damaged and fragile after training than before. 

RBC aggregation was shown to be altered in SCA patients which might also contribute to the pathophysiology of this disease [64,65]. RBC aggregation was shown to be partly related to plasma fibrinogen levels [64], which was not measured herein but might also be related to oxidative stress in SCA [66]. Oxidative/nitrosative stress markers were only measured within RBCs and besides a reduction in nitrotyrosine levels, stress markers were not affected by intervention. RBC aggregation index did not significantly change from T0 to T1 but the strength of RBC aggregates decreased with training. The data suggest that 30% less shear stress was necessary to prevent RBC aggregation after the training program. This high reduction rate might be of relevance in vivo. For instance, Lamarre et al. [67] reported an association between increased RBC aggregates strength and the risk of acute chest syndrome in SCA children. Endurance athletes show reduced aggregation index [68] which suggests that in general, training might positively affect aggregation. Since no effect of the applied training program on RBC aggregation was observed, we suspect that the training volume and intensity was too low to affect this parameter and again recommend that study duration show be longer in future investigations.

## 5. Conclusions

Our pilot study demonstrated that a controlled mild regular training program (i.e., cycling exercise, low volume and intensity) improved ventilatory efficiency of SCA patients. Moreover, our findings showed that regular physical exercise reduced oxidative/nitrosative stress and the amount of circulating free hemoglobin, which are known to play a key role in the genesis of vascular dysfunction in SCA. Finally, life quality seemed to improve after training possibly because of higher self-confidence to increase daily activity levels. Thus, physical activities below VT 1 seem somewhat safe and beneficial for SCA patients. Further studies should involve a control group and should now test whether higher training volumes might also improve performance parameters and blood rheology and should focus on possible training effects in HU treated and non-treated patients.

## Figures and Tables

**Table 1:** Red blood cell parameters before (T0) and after training (T1).

Parameter	T0	T1
Hb [g/dL]	9.2 (2.2)	8.8 (1.6)
MCV [fl]	92.1 (13.8)	91.5 (11.9)
MCH [pg]	32.0 (5.8)	35.9 (9.6)
MCHC [g/dL]	36.9 (4.0)	39.9 (8.5)
Free hemoglobin concentration [µg/mL]	3.6 (2.5)	2.8 (1.9) **
Corresponding heme levels [µM]	0.22 (0.1)	0.17 (0.1) **

Data are mean (SD). Significant difference between T0 and T1: ** *p* < 0.010; MCV, mean corpuscular volume; MCH, mean corpuscular hemoglobin; MCHC, mean corpuscular hemoglobin concentration.
